# Gesture screening in young infants: Highly sensitive to risk factors for communication delay

**DOI:** 10.1111/1460-6984.13150

**Published:** 2025-01-21

**Authors:** Katie Alcock, Kerstin Meints, Caroline Rowland

**Affiliations:** ^1^ Department of Psychology Lancaster University, Fylde College Lancaster UK; ^2^ School of Psychology University of Lincoln Lincoln UK; ^3^ Language Development Department Max Planck Institute for Psycholinguistics Nijmegen The Netherlands

**Keywords:** communicative development inventories, gesture development, language development, motor development, socioeconomic status, vocabulary development

## Abstract

**Introduction:**

Children's early language and communication skills are efficiently measured using parent report, for example, communicative development inventories (CDIs). These have scalable potential to determine risk of later language delay, and associations between delay and risk factors such as prematurity and poverty. However, there may be measurement difficulties in parent reports, including anomalous directions of association between child age/socioeconomic status and reported language. Findings vary on whether parents may report older infants as having smaller vocabularies than younger infants, for example.

**Methods:**

We analysed data from the UK Communicative Development Inventory (Words and Gestures); UK‐CDI (W&G) to determine whether anomalous associations would be replicated in this population, and/or with gesture. In total 1204 families of children aged 8–18 months (598 girls, matched to UK population for income, parental education and ethnicity as far as possible) completed Vocabulary and Gesture scales of the UK‐CDI (W&G).

**Results:**

Overall scores on the Gesture scale showed more significant relationships with biological risk factors including prematurity than did Vocabulary scores. Gesture also showed more straightforward relationships with social risk factors including income. Relationships between vocabulary and social risk factors were less straightforward; some at‐risk groups reported higher vocabulary scores than other groups.

**Discussion:**

We conclude that vocabulary report may be less accurate than gesture for this age. Parents have greater knowledge of language than gesture milestones, hence may report expectations for vocabulary, not observed vocabulary. We also conclude that gesture should be included in early language scales partly because of its greater, more straightforward association with many risk factors for language delay.

**WHAT THIS PAPER ADDS:**

**What is already known on the subject**

- We already know that it is possible to measure children's early communicative skills using parent‐completed inventories (Communicative Development Inventories, CDIs) and that some aspects of early communication can predict which children are likely to go on to have long‐lasting communicative development difficulties. We also know that most uses of CDIs include only vocabulary, not early gesture. In addition, child‐related and family‐related variables such as prematurity, family history of language disorder and socioeconomic status may be related to communication development.

**What this paper adds to existing knowledge**

- We looked at a large sample (*N* = approx. 1200) of families representative of the UK population with an infant aged 8 through 18 months and asked them about their infant's comprehension and production vocabulary as well as their early gesture skills. Gesture was more closely related to possible risk factors for communication development difficulties, for example, family history of communication difficulties, prematurity and multiple birth status. Vocabulary was only related in a straightforward way to family history and had complex relationships to socioeconomic differences. Families with different economic backgrounds may approach questions about their child's development in different ways.

**What are the potential or actual clinical implications of this work?**

- We suggest that clinicians need to ensure that not only vocabulary but also gesture ability is assessed when looking at very early communication skills. We also suggest that gesture may be more predictive of later communicative development difficulties, and that clinicians need to be sure that parents are clear on how vocabulary questions are asked and what exactly is required of them in answering these types of questions.

## INTRODUCTION

### Association of language and gestural ability

Learning to communicate with gestures and with words goes hand in hand. Children learning a spoken language usually produce their first communicative gestures before they produce their first spoken words (Fenson et al., 1994). There is a long‐standing body of research closely linking these two forms of development (Alcock & Connor, 2021; Bates, 1980; Bates & Dick, 2002) and some difficulties with gesture are also found in children with spoken language impairments such as developmental language disorder (DLD; Botting et al., 2010; Wray et al., 2016). A meta‐analysis of the pointing gesture and its relationship to early language development found that both concurrently and longitudinally there is a very strong relationship between this first communicative gesture in particular and the emergence of language (Colonnesi et al., 2010). Producing an item in gesture often precedes producing that item as a spoken word, and the timing of onset of a child producing a gesture‐word combination also predicts the timing of onset for a two‐word combination (Iverson & Goldin‐Meadow, 2005); this phenomenon is also observed in other languages and cultural settings (Capirci et al., 1996; Jensen de López, 2010).

When measuring early communication however, many scales only assess vocabulary (comprehension and/or production). For example, Dale and Penfold (2011) surveyed adaptations of the MacArthur Bates Communicative Development Inventory (MB‐CDI; Fenson et al., 2007) from more than 50 languages, and reported that, of these, researchers from only 10 language groups mention the use of gestures as part of their scale, even though the original form of the MB‐CDI uses gestures as part of the communication assessment for the younger group of children, aged 8–18 months.

Yet this youngest group of children have few spoken words in comparison to their comprehension vocabulary. At 12 months, median comprehension vocabulary for US English speaking children on the MB‐CDI is 77 words out of 396, and median production vocabulary for the same sample at the same age is only 6 words: most analyses of early word production are confounded by floor effects. For gesture production, median at 12 months is 25 out of 63 items (19.4% for comprehension, 1.5% for word production and 39.7% for gesture production respectively) (Frank et al., 2019). Scales that ignore gesture production are missing important information about children's early productive communication.

Likewise, the gesture assessment in the original MacArthur CDI (Fenson et al., 1994) was not fully validated against external measures of gesture, though this was completed in the second edition, now termed MacArthur‐Bates CDIs (MB‐CDIs, Fenson et al., 2007) and in the UK‐CDI (W&G) (Alcock et al., 2020). Further work on communicative, symbolic and meaningless gesture in UK toddlers has been carried out, using a variety of measures, but this is largely laboratory administered and hence not amenable to use for large‐scale screening (Alcock & Krawczyk, 2010).

### Importance of gesture in predicting language ability and language delay

Studying early gestures can also, potentially, provide important information about later language delay. Early word comprehension is reasonably predictive of which delayed talkers go on to catch up with their peers (Chilosi et al., 2019; Fisher, 2017; Thal et al., 1991). Fisher's (2017) review and meta‐analysis is particularly helpful here, as it summarises and grades the effects of different factors in predicting ongoing expressive language delay. The best predictor of language delay outcome across studies was indeed language comprehension. However, these studies have by and large not examined the relationship of early communicative gesture to language outcomes. If we do not include gesture as well as comprehension in studies of early language outcomes, we cannot be sure that it is only comprehension that predicts later language delay.

Indeed, gesture is also closely related to, and predictive of, future language in children with and without delay (Thal et al., 1991). Where gesture has been included in the precursors that a study investigates, it has been found to be highly predictive of vocabulary outcomes for up to a year later (Bavin et al., 2008). In this longitudinal study, Bavin et al. discuss ‘gesture’ and object use’ which is what we here are referring to as ‘gesture’, measured using the Australian version of the MB‐CDI. While Bavin et al. found that younger (8‐month) gesture was not as predictive of 12‐ and 24‐month language as older (12‐month) gesture, Cadime et al. (2017) found that both 9‐ and 12‐month gesture were predictive of later infant vocabulary. Luke et al. (2019) further found that the onset of pointing predicted language ability at preschool ages (3 and 4 years).

Interestingly, communicative gesture at 12 and 24 months has also been found to be the most predictive variable for diagnosis of autism spectrum disorder (ASD) aged 4 (Veness et al., 2012). Spontaneous gesture at 12 months was predictive of an ASD diagnosis and receptive language up to 2 years later (Choi et al., 2020). In particular, deictic gesture (pointing, reaching, showing) is delayed in children with ASD and some differences have been found in children at‐risk for ASD (younger siblings of children with an ASD diagnosis) who went on to receive an ASD diagnosis versus those who did not (Manwaring et al., 2018).

### Risk factors for language delay

Here we take a first step in studying whether a validated, normed, CDI gesture scale has potential to predict spoken language and perhaps even language delay, by investigating whether it is sensitive to the risk factors that we generally find to be predictive of language delay. We analysed gesture data from the open access dataset UK‐CDI W&G (Alcock et al., 2020; Alcock et al., 2020) and risk factor data based on Rudolph's (2017) meta‐analysis of factors assessed to determine whether these are associated with language delay, both biological and social. Rudolph (the only meta‐analysis of pre‐infancy and infant risk factors for DLD that we are aware of) included all factors that more than one previous study had examined (whether found significant or not) and of those the following are available in the UK‐CDI (W&G) dataset:

Biological risk factors: family history of language delay, child sex, prematurity.
Social risk factors: maternal education, birth order, parent marital status.

In addition, the dataset contains the following potential risk factors that were not examined by Rudolph: family income, paternal education, maternal age, birthweight, multiple birth status. These factors are also included because at least one study has reported an association between these variables and later language outcomes (Law et al., 2019 looked at household income; Pan et al., 2004 looked at the relationship between maternal age and child language ability; Reilly et al., 2006 found that multiple birth status was associated with poor language outcomes; Stanton Chapman et al., 2002 found that very low birthweight was associated with language impairment; Tomblin et al., 1997 like many studies looked at both parents' education together).

The first aim of the present study was to investigate whether a number of potential risk factors that are known to be associated with language delay are also associated with gesture use as measured by the UK‐CDI (W&G) (Alcock et al., 2020). We hypothesised that the greater sensitivity of the gesture scale to differences in the age group 8–18 months would mean that this scale can differentiate better between children who are exposed versus not exposed to these potential risk factors.

### Accuracy and anomalies in parent report of children's communicative development

**FIGURE 1:**
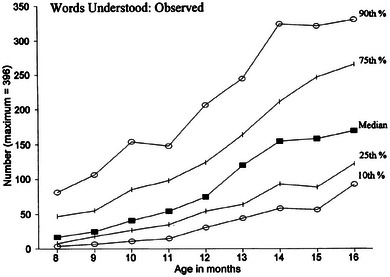
Median and centile vocabulary comprehension scores for each month of age, from Fenson et al. (1994).

A second aim of the study was to determine whether gesture report may be susceptible to fewer demand characteristics than vocabulary report. Several studies have noted anomalies in parent report of their children's language development, in particular, that may lead to problems with use of parent reported language as a predictor of later development. Fenson et al. (1994) noted that parent report of infants’ vocabulary comprehension did not smoothly increase with the infant's age – Figure 1 shows their observed data from the US MB‐CDI (W&G) for Words Understood.

Fenson et al. (1994, p. 36) further state that the number of words reported as understood by some of the youngest group of children was much higher than any previous literature might predict. They commented that parents may be assuming a level of comprehension that the children do not genuinely have. It is of course true that parents may struggle to understand what ‘comprehending’ a word may look like for the smallest children, and it is certainly not as easy to identify if a child understands a word as it is to identify whether they produce a word or gesture, though note that Feldman et al. (2000) found that some measures of toddler language production (particularly grammatical abilities) as measured on the MB‐CDI Words and Sentences (MB‐CDI W&S) also showed variable patterns of difference between risk groups, suggesting that the reporting error is not solely restricted to the reporting of word comprehension.

The so‐called reporting bias in parental reports of early word development seems to be related to socioeconomic status (SES). Reznick (1990) also found anomalies relating to parent SES in parent report of comprehension vocabulary. For example, for boys only, parents with higher SES reported lower levels of vocabulary, which is opposite to the expected direction based on risk of language delay. He suggested that parents with lower SES may be inclined to overestimate children's language abilities. However, it is also possible, of course, that higher SES parents may under‐report children's language abilities, perhaps if they spend less time with their child so have fewer data points to observe. It is true that on the whole families on higher incomes work more hours, but the relationship for the UK is far from straightforward (Eiser et al., 2021).

Interestingly, Tamis‐LeMonda et al. (2002) reported that lower income mothers tended to assume a younger age of onset of milestones, and this and other work shows that knowledge of language milestones is more accurate than other domains of development (Tamis‐LeMonda et al., 1998; Tamis‐LeMonda et al., 2002). These include domains that are at least as easily observable as gesture or language production, such as cognition related behaviours. One possible explanation of is therefore that parental knowledge of expected milestones may lead to *inaccurate* reporting of children's skills. Parents have been reported to anticipate infants’ behaviour based on their knowledge of milestones: Hendrix and Thompson (2011) found mothers used more prohibitions and set up more baby‐proofing when they anticipated their child's onset of locomotion rather than after the fact. Gesture milestones, on the other hand, are not as widely known by parents as language milestones (Sidman, 2018) and therefore less likely to be anticipated. Of course, parent knowledge of child development changes over time and this state of knowledge may not be the same in future generations of parents.

The second aim of the present study was to determine if parent report of gesture is more straightforwardly related to risk factors and to age than parent report of either comprehension or production of single words, which might both be more affected by reporting bias than gesture report. Because all of the risk factors on which data were collected existed before language and communication data were collected, we can here determine whether these risk factors more straightforwardly predict communicative development at our measurement time point.

In conclusion, we aimed to determine whether using gesture report will improve measurement properties and potential for prediction of later language delay of the UK‐CDI, compared to parent report of vocabulary production and comprehension in this sample of UK families (Alcock et al., 2020). We hypothesised that potential greater sensitivity of the gesture scale to differences in the age group 8–18 months will mean that this scale can differentiate better between children who are exposed to a variety of social and biological risk factors. We further hypothesised that parent report of gesture may be more straightforwardly related to demographic factors and to age than parent report of either comprehension or production of single words.

## METHODS

### Participants

#### Standardisation sample

A total of 1693 families were recruited from all regions and nations of the UK, using a variety of online, in person and other methods (Alcock et al., 2020). These 1693 families were those who completed a sufficient proportion of the UK‐CDI (W&G) to be included. Parents completed the UK‐CDI (W&G) either on paper or online and the inter‐form reliability was also validated for these two methods. From these, data from 1210 who had completed the full vocabulary questionnaire and were representative of the UK in terms of region/nation of residence, ethnic origin, SES, child disability and parental level of education, were extracted for the overall UK‐CDI (W&G) stratification sample (taking this information from the Family Questionnaire that accompanied the UK‐CDI (W&G)). Full information on the representative nature of the sample can be found in the UK‐CDI (W&G) Manual (Alcock et al., 2020), as well as full details of all the items on the UK‐CDI (W&G). For brevity, the subscales included in the Gestures section are labelled First Communicative Gesture, Games and Routines, Actions with Objects, Pretending to be a Parent and Imitating Other Actions, and are taken from the original MB‐CDI (W&G) (Fenson et al., 2007).

Of the full stratification sample of 1210, 1204 completed some or all questions on the Gesture subscale of the UK‐CDI W&G scale. From these, children's age and sex are shown in Table 1. As part of the Family Questionnaire, information was available in the database for biological and social risk factors. Biological: gestational age, birthweight, family history of language disorders in a first degree relative, the child's sex and multiple birth status. Social: maternal education, birth order, parent marital status, family income, paternal education, time spent in out‐of‐home care, current maternal age and index of multiple deprivation (IMD, a national rank but here coded in quintiles; Office for National Statistics, 2004). Some parents (those on low incomes and belonging to groups that were originally under‐sampled) were given £5 supermarket vouchers as a thank you for participation; further information on this, and on participation rates, are available in Alcock et al. (2020). The full standardisation dataset is available in open‐access format on the UK Data Archive (Alcock et al., 2020). The study design was not preregistered.

**TABLE 1:** Number (by age and sex) of children in families who attempted or completed the gesture subscale.

Child's age in completed months	Number of girls	Number of boys	Total (*N*)
8	52	45	97
9	64	65	129
10	52	68	120
11	54	49	103
12	56	58	114
13	59	56	115
14	49	48	97
15	63	49	112
16	60	60	120
17	51	51	102
18	38	57	95
Total completing gesture scale	598	606	1204

### Materials

Full details of the UK‐CDI (W&G) are available in Alcock et al. (2020). Briefly, it consists of a checklist of 395 words in 19 subsections (e.g., animal words, verbs) and 63 gestures in 5 subsections (e.g. ‘communicative gestures’, ‘actions with objects’). Parents endorse whether their child can say and understand, or just understand, each word, with instructions to leave words blank that the child does not say or understand. Parents are instructed to select whether their child uses each gesture—‘Not Yet, Sometimes or Often’ for the sub‐section ‘communicative gestures’, and to select ‘Yes or No’ for the remaining sub‐sections.

Following completion of the UK‐CDI (W&G), parents were asked to complete a demographics questionnaire, the Family Questionnaire. This included questions on the child's health, diagnosed disabilities, family education and occupation, household and house size, income, childcare, exposure to languages other than English and family history of language disability. Household income was divided into the bands 1: ‘0 to £14000’, 2: ‘14001 to £24000’, 3: ‘£24001 to £42000’ and 4: ‘£42001 and higher’, representing, at the time of data collection, incomes below the poverty level, above the poverty level but below median income, above median income but below the higher rate tax bracket and above the higher rate tax bracket. Again, full details are available in Alcock et al. (2020).

### Procedure

#### Scoring

From the UK‐CDI (W&G), the total number of words that the parent endorsed as ‘understands and says’ was used as the child's production vocabulary score (this is a single point for each item, and is a standard question for the CDI (W&G) in all languages; the child must say the word to receive this point). The total number endorsed as either ‘understands’ or as ‘understands and says’ was used as the child's comprehension vocabulary score. The child's gesture score was calculated by multiplying 0.5 by the child's score for the Communicative Gestures subscale (which had possible scores of 0, Not yet, 1, Sometimes or 2, Often for each item), summed with total number of all other gesture items endorsed Yes by the parent (which only had possible scores of 0, No, or 1, Yes).

#### Ethics information

All parents gave informed consent for participation with ethics approval from Lancaster University Research Ethics Committee or, for the validation sample, University of Lincoln Research Ethics Committee. Further ethics information is available in Alcock et al. (2020).

## RESULTS

### UK‐CDI: Relationship between gesture and vocabulary scores and age

**FIGURE 2:**
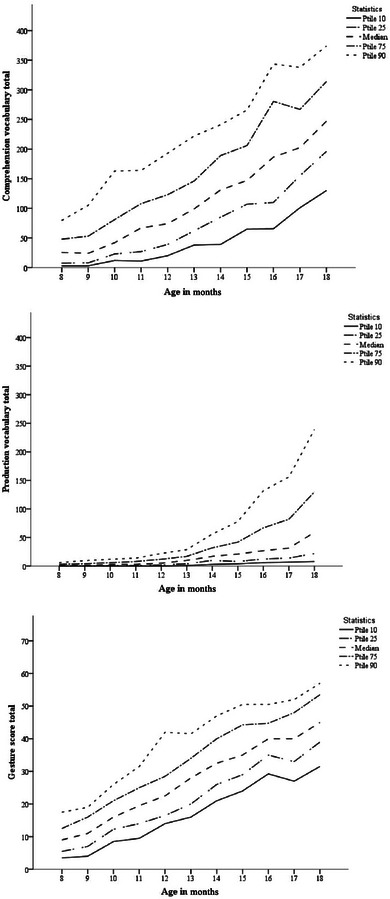
Median and centile vocabulary (comprehension followed by production) and gesture scores for each month of age, from Alcock et al. (2020).

Correlations between children's total gesture score and total number of words produced and comprehended are shown in Table 2. There were medium to large significant correlations (all *r* > 0.40, all *ps* < 0.001) between all subscales of the UK‐CDI (W&G) and with child age. Mean UK‐CDI (W&G) vocabulary comprehension, vocabulary production and gesture scores for each month of age can be seen in Figure 2.

**TABLE 2:** UK‐CDI (W&G) relationships between gesture, production and comprehension vocabulary and age.

Variable	*n*	1	2	3
1. Gesture total	1204	–		
2. Production vocabulary	1210	0.56 (<0.001)	–	
3. Comprehension vocabulary	1210	0.78 (<0.001)	0.67 (<0.001)	
4. Age	1210	0.79 (<0.001)	0.48 (<0.001)	0.67 (<0.001)

*Note*: Cells show *r* (*p*).

### Associations of parent‐reported gesture and vocabulary scores with risk factors

#### Biological risk factors, vocabulary and gesture

For biological risk factors in the dataset (gestation, birthweight, presence of language disorder in a first degree relative, sex and multiple birth status), we examined association with vocabulary (production and comprehension) and gesture scores, controlling for child's age. These variables were all either inherently categorical (e.g., child sex) or coded as categorical (e.g., gestation at birth was classified into 33 weeks or fewer, 34–36 weeks and 37 weeks or more). Analyses of covariance (ANCOVAs) were carried out with child age as covariate.

For variables with more than two groups where a significant main effect was found, we set an intent that post‐hoc comparisons between groups would be carried out, restricting these to the analyses that showed a significant main effect, and using the Holm‐Bonferroni method to correct for multiple comparisons. All group comparisons, regardless of whether a main effect was found, are shown in Figures 3, 4, 5, 6, 7, 8, 9, 10, 11, 12, 13, 14, 15.

**FIGURE 3:**
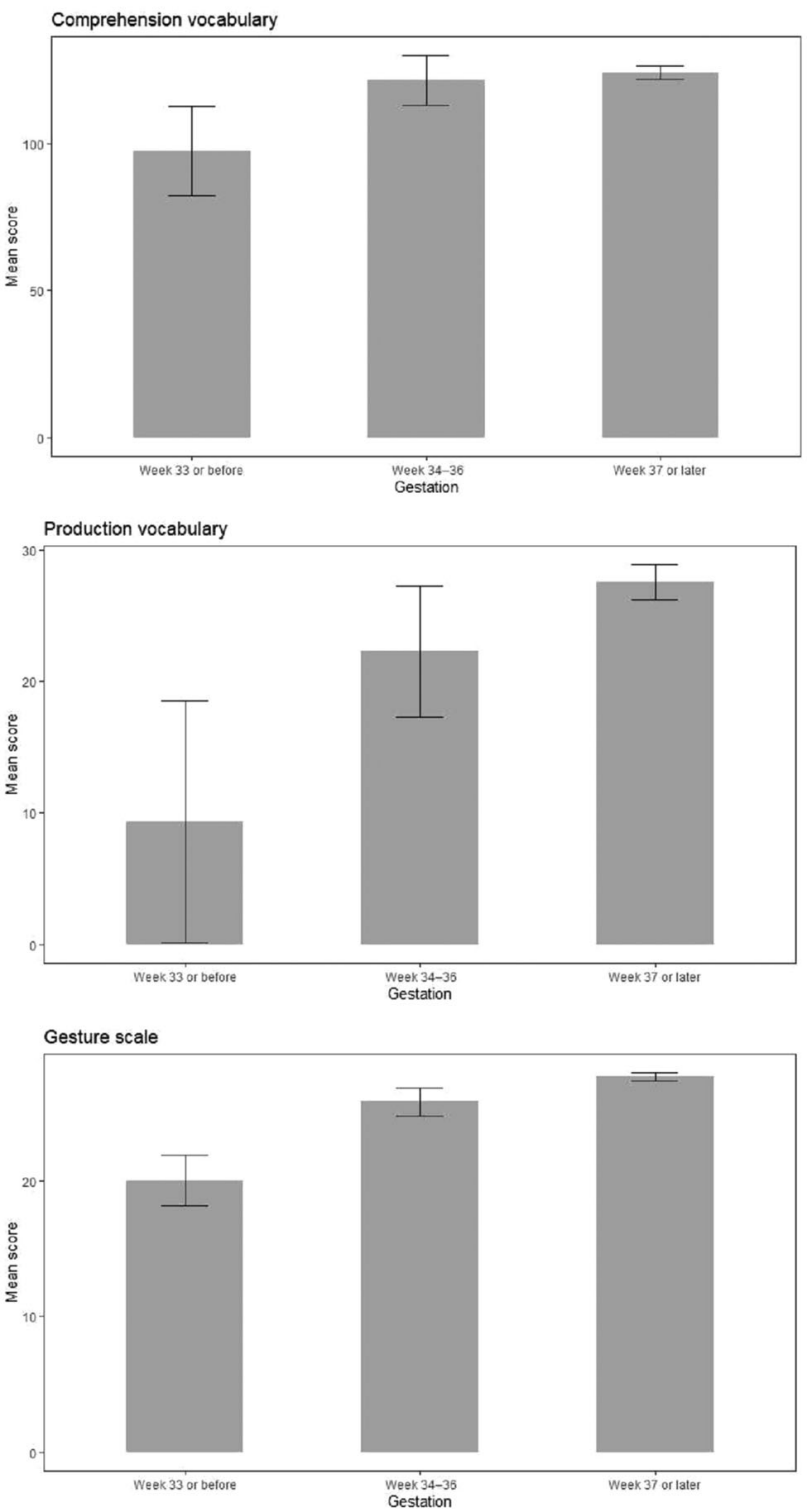
Vocabulary and gesture scores for children born at different gestational ages.

**FIGURE 4:**
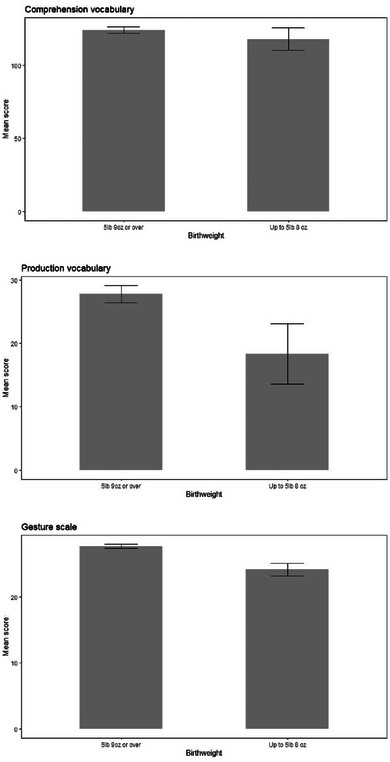
Vocabulary and gesture score for children of different birthweights.

**FIGURE 5:**
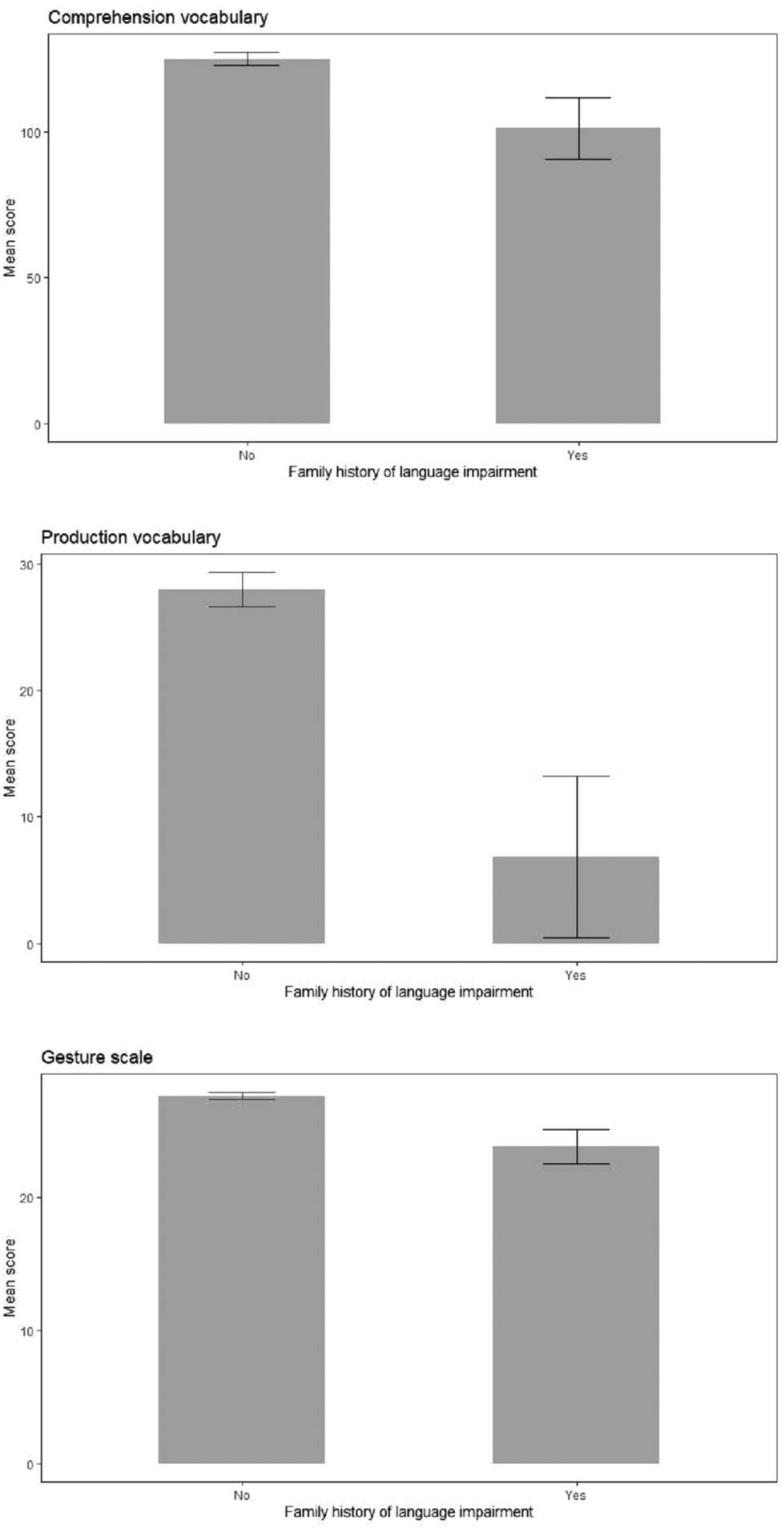
Vocabulary and gesture score for children with and without an immediate family history of language disorder.

**FIGURE 6:**
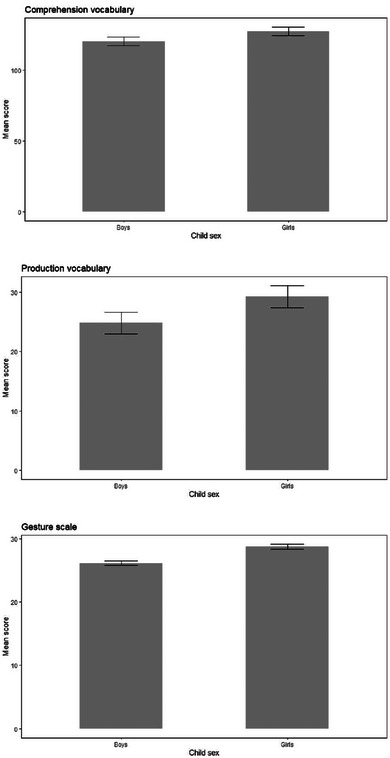
Vocabulary and gesture score for boys and girls.

**FIGURE 7:**
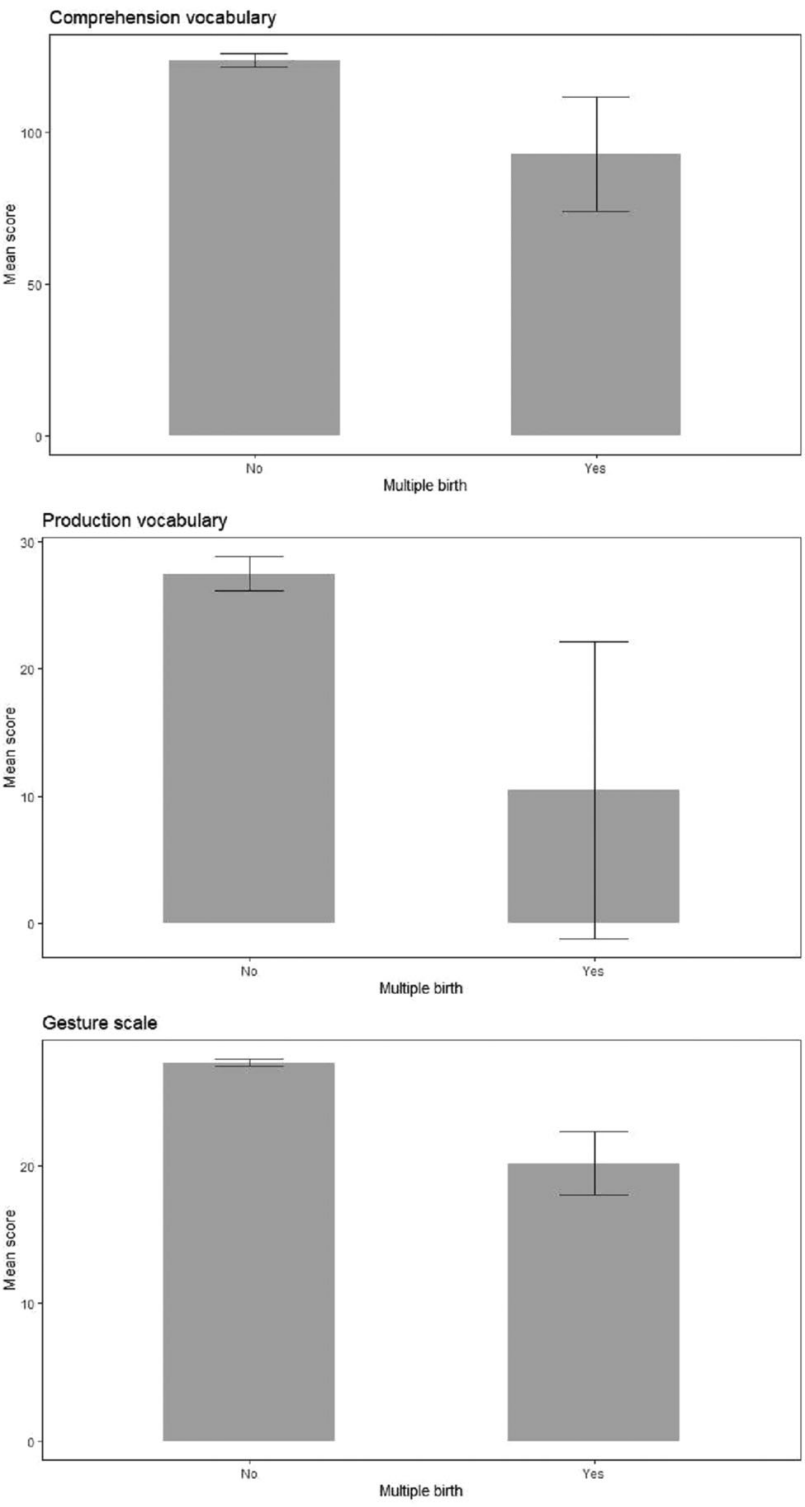
Vocabulary and gesture score for children who are singletons or from multiple births.

**FIGURE 8:**
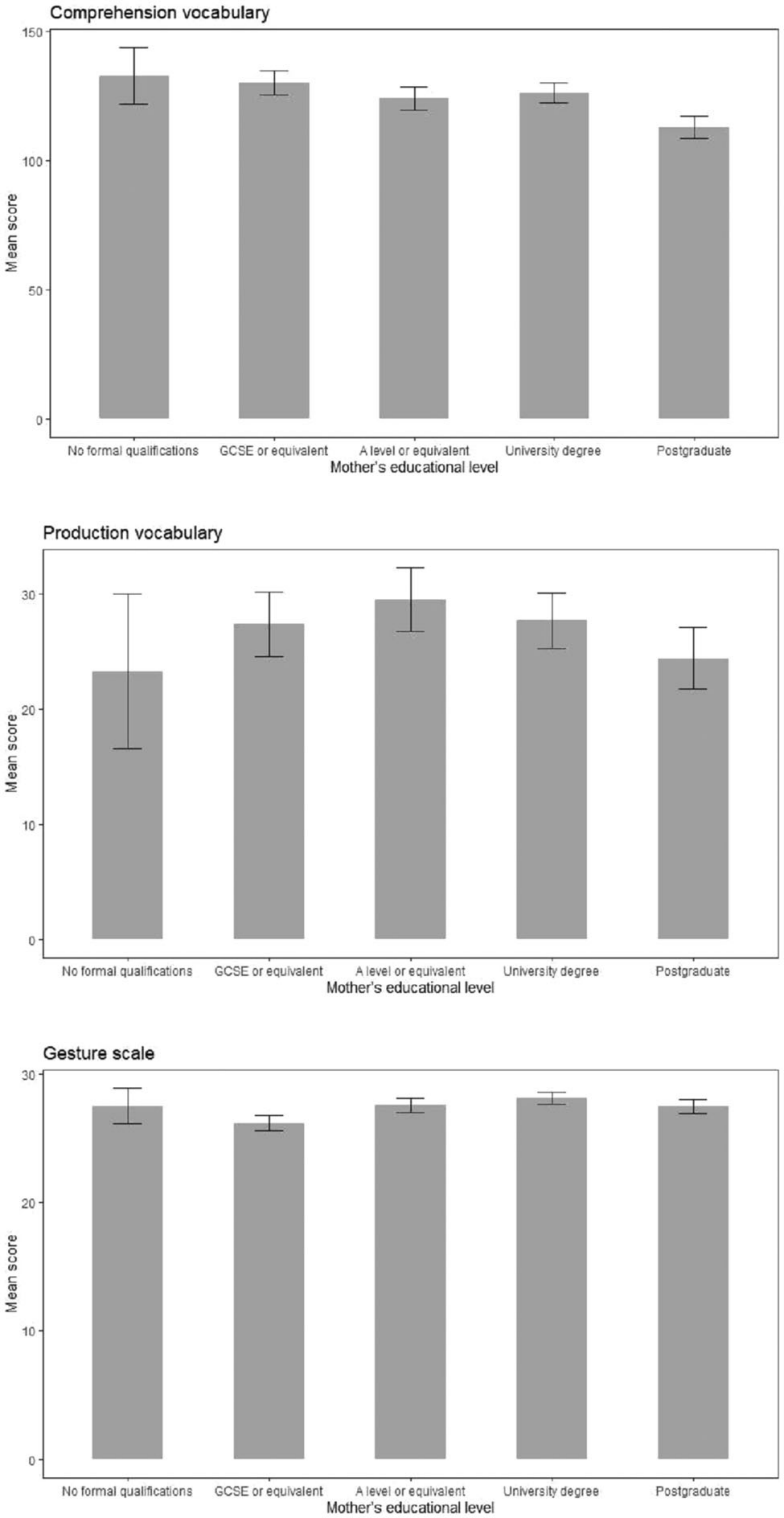
Vocabulary and gesture score for children whose mothers have different educational levels. Abbreviation: GCSE, General Certificate of Secondary Education.

**FIGURE 9:**
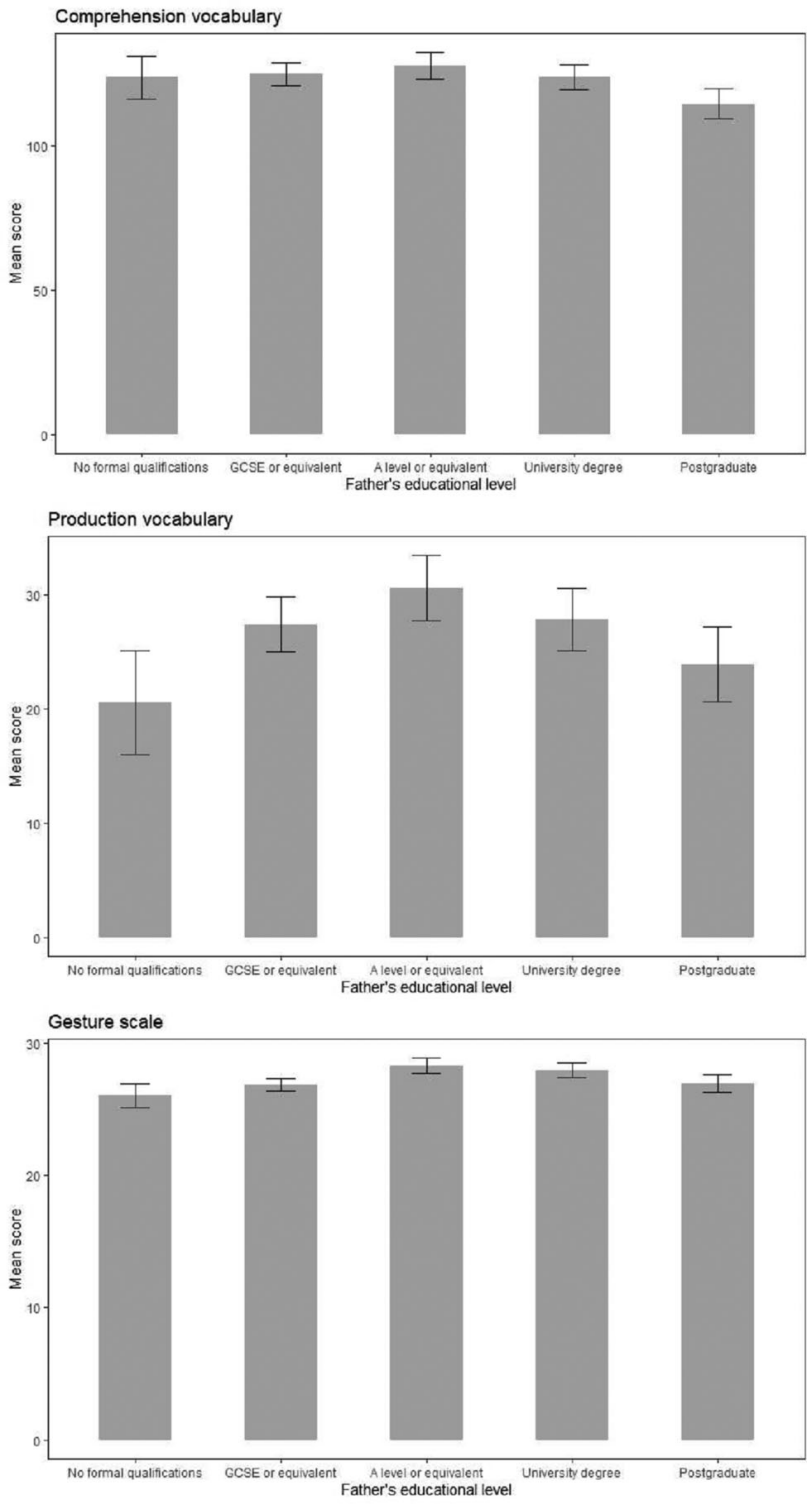
Vocabulary and gesture score for children whose fathers have different educational levels. Abbreviation: GCSE, General Certificate of Secondary Education.

**FIGURE 10:**
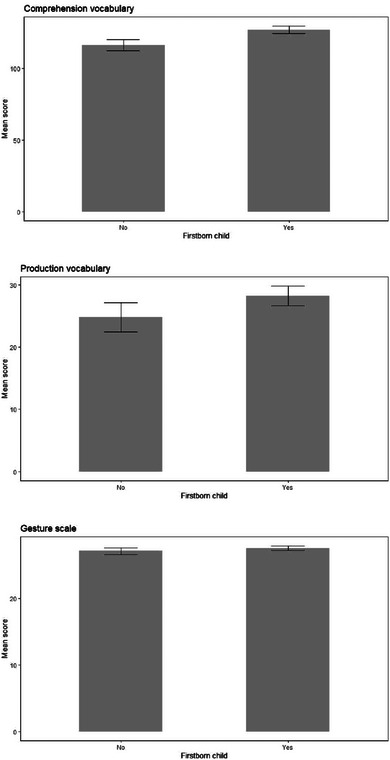
Vocabulary and gesture score for children who are firstborn or younger children.

**FIGURE 11:**
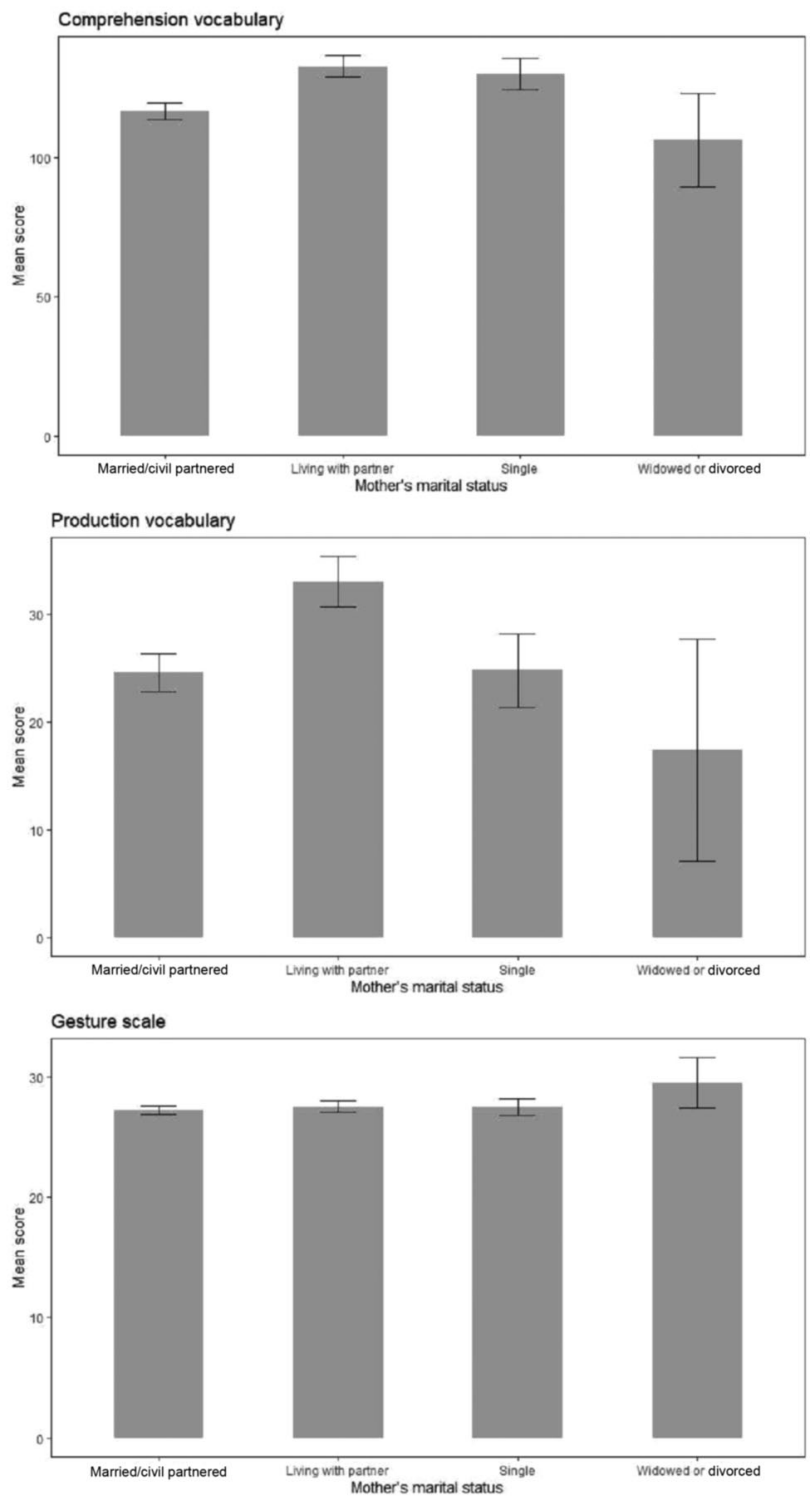
Vocabulary and gesture score for children whose mothers have different marital statuses.

**FIGURE 12:**
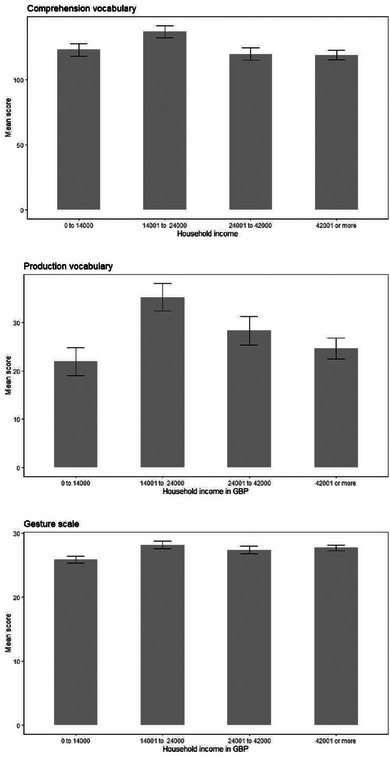
Vocabulary and gesture score for children from households with different income levels.

**FIGURE 13:**
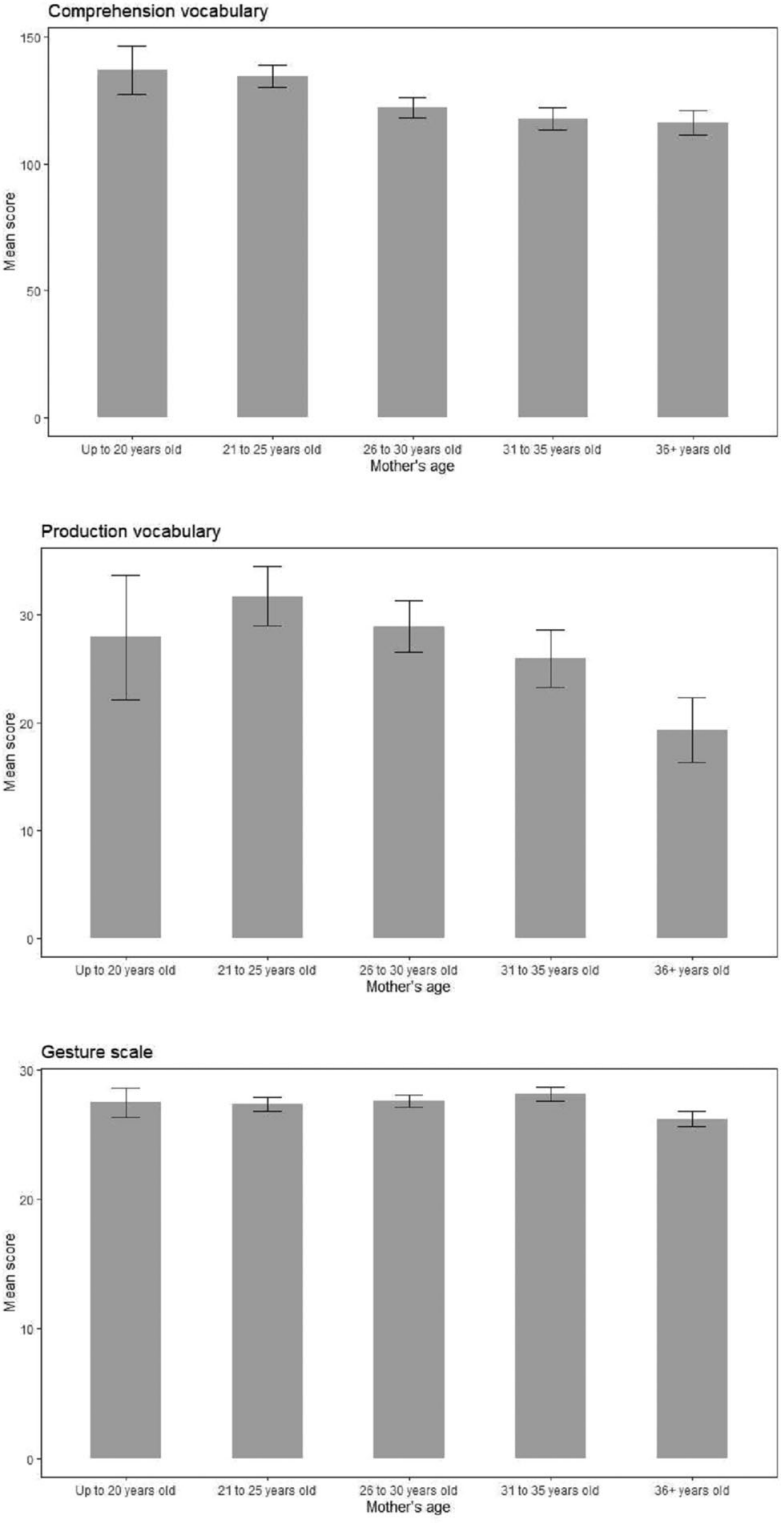
Vocabulary and gesture score for children whose mothers are different ages.

**FIGURE 14:**
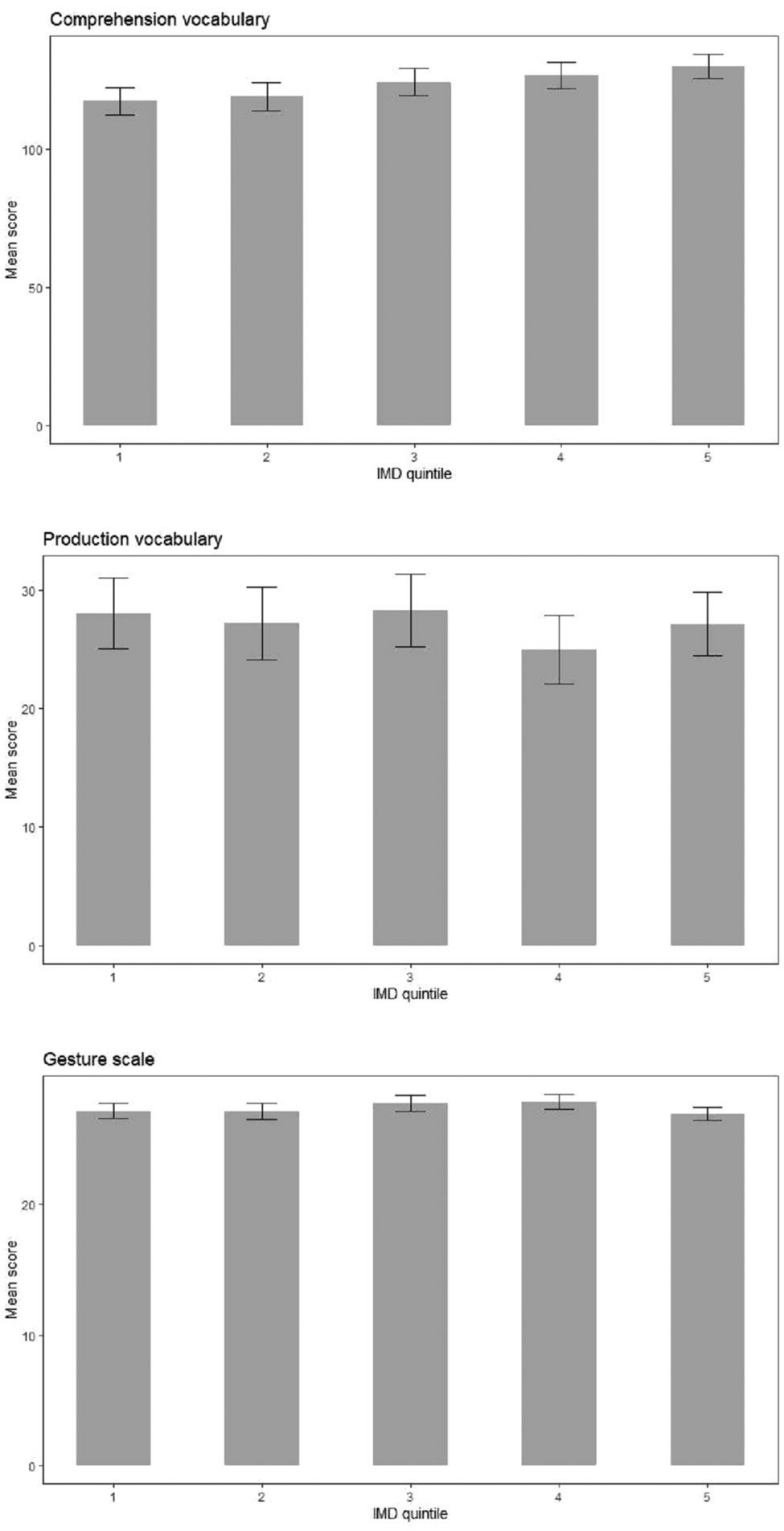
Vocabulary and gesture scores for children whose families live in areas with different indices of multiple deprivation, where Quintile 1 is more deprived and Quintile 5 is less deprived. Abbreviation: IMD, index of multiple deprivation.

**FIGURE 15:**
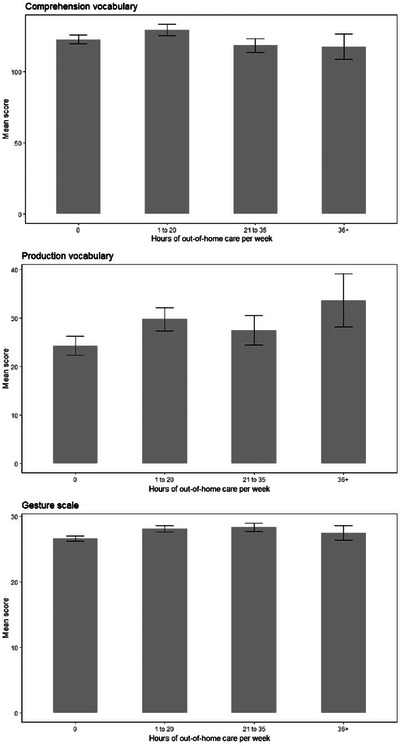
Vocabulary and gesture scores for children with different experiences of out‐of‐home care.

Results of these ANCOVAs, and group means, are reported in Table 3. Gesture is significantly associated with all risk factors while comprehension and production vocabulary are only significantly associated with family history of a language disorder. Where totals do not tally with the total number of questionnaires this is due to parents choosing not to answer a question.

**TABLE 3:** Analyses of covariance (controlling for child age) for the association of biological risk factors with comprehension vocabulary, production vocabulary and gesture score.

Risk factor	Measure	EMM	SE	EMM	SE	EMM	SE	df	F	η	*p*
		Subgroup (*N*)									
		Extremely/very preterm (23)	Preterm (78)	Term (1095)							
Child gestational age	Comprehension vocabulary	97.63	15.35	121.69	8.34	124.27	2.22	2, 1192	1.50	0.003	0.22
	Production vocabulary	9.33	9.24	22.27	5.02	27.54	1.34	2, 1192	2.35	0.004	0.10
	Gesture score	20.00 (from term, *p* < 0.001)	1.87	25.80 (from very preterm, *p* < 0.001)	1.02	27.66 (from preterm, *p* > 0.05)	0.27	2, 1192	9.50	0.016	<0.001
		Subgroup (*N*)									
		Low or very low (89)		Normal (1113)							
Child birthweight	Comprehension vocabulary	117.98	7.81	124.07	2.21			1, 1199	0.56	<0.001	0.45
	Production vocabulary	18.35	4.76	27.74	1.34			1, 1199	3.61	0.003	0.06
	Gesture score	24.12	0.95	27.64	0.27			1, 1199	12.66	0.010	<0.001
		Subgroup (*N*)									
		No (1148)		Yes (49)							
Family history of language disorder	Comprehension vocabulary	124.89	2.17	101.16	10.52			1, 1194	4.883	0.004	0.027
	Production vocabulary	28.02	1.32	6.84	6.40			1, 1194	10.49	0.009	0.001
	Gesture score	27.61	0.26	23.80	1.28			1, 1194	8.46	0.007	0.004
		Subgroup									
		Boy (606)		Girl (598)							
Child sex	Comprehension vocabulary	120.21	2.99	127.16	3.01			1, 1201	2.69	0.002	0.10
	Production vocabulary	24.85	1.82	29.25	1.83			1, 1201	2.90	0.002	0.09
	Gesture score	26.10	0.36	28.69	0.37			1, 1201	25.26	0.021	<0.001
		Subgroup (*N*)									
		Singleton (1158)		Multiple birth (15)							
Multiple birth status	Comprehension vocabulary	123.79	2.15	92.70	18.87			1, 1170	2.68	0.002	0.10
	Production vocabulary	27.47	1.33	10.48	11.68			1, 1170	2.09	0.002	0.15
	Gesture score	27.49	0.26	20.18	2.31			1, 1170	9.85	0.008	0.002

*Note*: Showing EMM, SE and, where relevant, significance of post‐hoc comparisons as well as the number of comparisons for which post‐hoc pairwise comparisons were corrected.

Abbreviation: EMM, estimated marginal means.

For child gestation, post‐hoc comparisons were carried out with Holm‐Bonferroni corrections. Children born at 33 weeks or earlier (Extremely or Very preterm, *N* = 23) had significantly lower gesture scores than both children born between 34 and 36 weeks (Preterm) and those born after 36 weeks (Term), corrected for 4 comparisons. Mean differences, Ns, SE and *p* values are also shown in Table 3. Group comparisons are shown in Figure 3.

Children with a lower birthweight, children with a family history of language disorder, boys and twins/multiples also had significantly lower gesture scores. Only children with a family history of language disorder had significantly lower production and comprehension vocabulary than those without a family history. All of these comparisons are shown in Figures 3, 4, 5, 6.

In summary, children in categories that are considered to be of higher biological risk for language disorder have lower gesture scores in five out of five comparisons. Only children in the high‐risk family history category also have lower comprehension scores and production scores.

### Social risk factors, vocabulary and gesture

We investigated which social risk factors (taken from Rudolph, 2017, or available in the dataset) have a significant association with vocabulary (production and/or comprehension) and gesture scores, controlling for the child's age. These are maternal education, birth order, parent marital status, family income, paternal education, time spent in out‐of‐home care, maternal age and IMD.

These were coded as categorical variables; for example, maternal age was coded from 1 (up to 20 years old) to 5 (36 years or older). ANCOVAs showing the results of these comparisons can be seen in Table 4, with age as covariate. Post‐hoc comparisons between groups where more than two categories are present were carried out but only where a main effect was seen. Main effects are shown in Table 4 as are estimated marginal means, mean differences, SEs and *p* values. These comparisons reported are corrected using the Holm‐Bonferroni correction, analysis‐wise.

**TABLE 4:** Results of ANCOVAs (controlling for age) analysing the effect of social risk factors on reported comprehension vocabulary, production vocabulary and gesture score.

Risk factor (multiple comparisons)	Measure	EMM	SE	EMM	SE	EMM	SE	EMM	SE	EMM	SE	df	F	η	p
		Subgroup (N)													
		No formal qualifications (45)		GCSE ‐/similar (251)		A level/similar (269)		University degree/similar (345)		Postgraduate/similar (282)					
Mother's education	Comprehension vocabulary	132.72	10.94	13.09	4.63	124.22	4.47	126.39	3.95	112.92	4.37	4, 1186	2.29	0.008	0.06
	Production vocabulary	23.3	6.72	27.4	2.85	29.53	2.75	27.73	2.43	24.41	2.68	4, 1186	0.55	0.002	0.70
	Gesture score	27.51	1.35	26.17	0.57	27.53	0.55	28.12	0.49	27.49	0.54	4, 1186	1.74	0.006	0.14
		Subgroup (N)													
		No formal qualifications (98)		GCSE/similar (347)		A level/similar (253)		University degree/similar (274)		Postgraduate/similar (190)					
Father's education	Comprehension vocabulary	123.8	7.42	125.13	3.94	127.76	4.63	123.89	4.45	114.65	5.33	4, 1156	0.95	0.003	0.44
	Production vocabulary	2.55	4.55	27.4	2.42	30.59	2.84	27.79	2.73	23.91	3.27	4, 1156	1.16	0.004	0.33
	Gesture score	26.02	0.91	26.85	0.48	28.28	0.57	27.95	0.55	26.95	0.65	4, 1156	1.86	0.006	0.12
		Subgroup (N)													
		Younger (372)		Firstborn (823)											
Birth order	Comprehension vocabulary	116.34	3.82	126.94	2.57							1, 1192	5.29	0.004	0.022
	Production vocabulary	24.81	2.33	28.21	1.57							1, 1192	1.46	0.001	0.22
	Gesture score	27.06	0.47	27.51	0.31							1, 1192	0.62	0.001	0.43
		Subgroup (N)													
		Married/civil partnered (628)		Living with partner (373)		Single (171)		Divorced, separated or widowed (19)							
Mother's marital status (6 comparisons)	Comprehension vocabulary	116.68	2.92	132.87 (from married, p = 0.001)	3.78	130.20 (from married, p = 0.032)	5.59	106.21	17.22			3, 1186	4.70	0.012	0.003
	Production vocabulary	24.56 (from partnered, p = 0.004)	1.79	32.99	2.33	24.75 (from partnered, p = 0.047)	3.43	17.34	10.58			3, 1186	3.26	0.008	0.021
	Gesture score	27.23	0.36	27.53	0.47	27.49	0.69	29.5	2.13			3, 1186	0.44	0.001	n.s.
		Subgroup (N)													
		Band 1£0 ‐ £14000 (241)		Band 2£14001 ‐ £24000 (254)		Band 3£24001 ‐ £42000 (236)		Band 4£42001 or more (426)							
Household income (6 comparisons)	Comprehension vocabulary	122.92	4.72	136.71	4.61	119.67 (from band 2, p = 0.05)	4.77	118.90 (from band 2, p = 0.012)	3.55			3, 1152	3.50	0.009	0.015
	Production vocabulary	21.92 (from band 2, p = 0.006)	2.89	35.23	2.82	28.31	2.92	24.61 (from band 2, p = 0.015)	2.17			3, 1152	4.34	0.011	0.005
	Gesture score	25.89 (from band 2, p = 0.024)	0.57	28.19	0.56	27.4	0.58	27.73	0.43			3, 1152	3.16	0.008	0.024
		Subgroup (N)													
		Up to 20 years old (60)		21 to 25 years old (267)		26 to 30 years old (352)		31 to 35 years old (280)		36+ years old (223)					
Mother's age (10 comparisons)	Comprehension vocabulary	136.93	9.44	134.58	4.47	122.3	3.90	117.7	4.37	116.18	4.90	4, 1176	3.05	0.01	0.016
	Production vocabulary	27.97	5.78	31.77 (from 36+, p = 0.02)	2.74	28.93	2.39	25.96	2.68	19.33	3.00	4, 1176	2.59	0.009	0.035
	Gesture score	27.48	1.16	27.37	0.55	27.62	0.48	28.11	0.54	26.21	0.60	4, 1176	1.46	0.005	0.21
		Subgroup (N)													
		1 (most deprived) (232)		2 (214)		3 (221)		4 (246)		5 (least deprived) 285)					
IMD quintile	Comprehension vocabulary	117.16	4.82	118.74	5.02	123.95	4.94	126.56	4.68	129.59	4.36	4, 1192	1.25	0.004	0.29
	Production vocabulary	28.04	2.96	27.16	3.08	28.29	3.03	24.95	2.87	27.13	2.67	4, 1192	0.20	0.001	0.94
	Gesture score	27.18	0.59	27.14	0.62	27.78	0.61	27.87	0.58	26.97	0.54	4, 1192	0.50	0.002	0.74
		Subset													
		0 h (534)		1 to 20 h (360)		21 to 35 h (223)		36+ h (69)							
Time spent in out of home care (6 comparisons)	Comprehension vocabulary	122.8	3.18	129.53	3.86	118.54	4.94	117.72	8.82			3, 1181	1.30	0.003	0.27
	Production vocabulary	122.8	1.96	129.53	2.38	118.54	3.04	117.72	5.43			3, 1181	1.58	0.004	0.19
	Gesture score	26.60 (from 1–20 h, p = 0.03)	0.39	28.1	0.47	28.32	0.61	27.46	1.08			3, 1181	2.86	0.007	0.036

*Note*: Showing EMM, SE and where appropriate, the number of comparisons corrected for in post‐hoc pairwise comparisons, together with their significance.

Abbreviations: EMM, estimated marginal means; GCSE, General Certificate of Secondary Education; IMD, index of multiple deprivation.

There were no significant differences on any vocabulary or gesture measure for children whose mothers or fathers were of different educational levels. These data are shown in Figures 7 and 8.

Firstborn children had significantly higher comprehension vocabulary than later borns but there were no significant differences for production vocabulary or for gesture. These data are shown in Figure 9.

Vocabulary and gesture scores for children with different parental marital status, household income and maternal age can be seen in Figures 10, 11, 12, 13, 14.

In summary, contrary to expectations, children growing up in a potentially more risky environment based on predictions from the literature (household income below median, younger mothers rather than older; single or cohabiting rather than married parents) reported higher comprehension and/or production vocabulary scores. However, for gesture, where differences were found between social risk factor groups, these were in the predicted direction: children in higher risk groups (those who have no out‐of‐home care, families with lower incomes) had lower gesture scores.

## DISCUSSION

### Measurement properties of the UK‐CDI (Words and Gestures) subscales

We report here a new analysis of data from the UK‐CDI (W&G) (Alcock et al., 2020; Alcock et al., 2020), specifically looking at the properties of the Gesture subscale, which is not invariably included on parent report instruments measuring early language development. This dataset is one of the largest sets of child language data, especially if we look at those that include gesture. It also comes from a demographically diverse sample that represents the overall UK‐English speaking population. Previously, this gesture scale has been found to have good measurement properties including good internal reliability, significant correlation with the other subscales and with child's age. Good external validity of the symbolic subscale of the gesture scale has also been shown when tested against laboratory measures of symbolic gesture (Alcock et al., 2020). The median proportions of gestures on the inventory that parents report that their children can perform is highly comparable to results from comparable populations (Frank et al., 2019).

Comparing the UK data to previously collected US data, for children's increase in vocabulary and communicative gesture with age (Figure 2), a similar increase followed by a decrease in scores is found at around the same age as in the Fenson et al. (1994) data, for production vocabulary. In the UK‐CDI (W&G) data, median scores for vocabulary at 12 months of age were 74 words in comprehension (equivalent to 12% of words on the inventory), 5 words in production (1.2%) and a gesture score of 22.5 (35% of the possible gesture total). Comparing these to Fenson et al. (2007)’s data, in which scores are of a similar order of magnitude to the UK‐CDI (W&G) data, we can also see a similar dip in comprehension vocabulary report at around the same age (10–11 months) for CDI‐reported data, but not for lab‐collected vocabulary data. This is similar also to data shown in Figure 1 in the Introduction, taken from Fenson et al. (1994).

### Subscales of the UK‐CDI (Words and Gestures) in relation to risk factors

We analysed the properties of this standardised, parent‐completed inventory of children's early communicative abilities. We predicted that the scale would show differences between children in categories of risk for language difficulties, both biological and social.

Looking at some risk categories, we find this to be the case. Children who have some biological risk factors that may affect language and communication development are measured as having poorer communicative development on the UK‐CDI (W&G). Children born prematurely, or with low birthweight, boys and twins/higher multiples, are all shown to have poorer gesture scores than children not in these biological risk categories. Children with a family history (first‐degree relative) of a language or communication disorder have poorer comprehension, production and gesture scores.

We note that gesture scores appear to be more closely related to a wider range of these biological risk factors than the vocabulary scale. In this age group, production vocabulary is likely to be low and limited in range, so it is not likely to show sufficient variability in scores to be sensitive to some risk factors. However, comprehension vocabulary scores, often relied upon to give an indication of a child's risk (Fisher, 2017), have substantial individual variability, yet are not associated with most biological risk factors.

Moving to social risk factors, children's comprehension vocabulary is significantly related to birth order, mother's marital status, household income and mother's age. Similarly, production vocabulary is significantly related to mother's marital status, household income and mother's age. Gesture is significantly related to household income and time spent in out of home care. However, the pattern of relationships is not as simple as with biological risk factors: for vocabulary scores, looking at family social risk factors, in each case parents in one or two intermediate categories (single mothers or those living with a partner, mothers aged 21–25 and households with incomes in Band 2, £14001 to £24000) reported their infants had higher vocabulary scores than infants whose risk factors we would have hypothesised would place them less at risk (married mothers, older mothers and those with higher incomes). For gesture, however, higher incomes (as predicted) and moderate hours of out of home care, were associated with better scores on the UK‐CDI (W&G). Again, the gesture scale appears to be more related to factors found to be significant in previous studies than are the vocabulary scales.

### Results in unexpected directions, and null relationships, between risk factors and communicative outcomes

Most biological risk factors only showed significant relationships with gesture, not vocabulary, scores. As mentioned, production vocabulary is low, and has low variability, at this age. This may be why we see no significant relationship. Comprehension vocabulary scores, on the other hand, vary widely in this age group and do not show a floor effect in the same way that production vocabulary does.

Added to this, some relationships were found between communicative outcomes and risk factors that were in an unexpected direction. These were all between comprehension or production vocabulary and social risk factors, such as when children of mothers in their 20s were reported to have higher vocabulary than older mothers, and mothers in a lower income bracket reported their children to have higher vocabulary than those in higher brackets.

It is difficult to explain these findings, though they also resemble Fenson et al. (1994)’s results, and findings from the main UK‐CDI (W&G) analysis (Alcock et al., 2020): Parents of infants of some younger ages report higher vocabulary scores than those of older infants, and Feldman et al. (2000)’s also found some unexpected directions of relationship between parent report of grammatical development and parent SES. These findings are generally mixed in direction and magnitude, and do not constitute a conclusive set of findings. Instead, they are a puzzle we may be able to solve in the future.

For this puzzle, we get some clues from previous findings on parental expectations and knowledge in different domains of child development. Both Tamis‐LeMonda et al. (1998) and Sidman (2018) found that parental knowledge of language milestones allies more closely to empirical data than that of cognitive or gesture milestones respectively.

Further, knowledge of the pointing milestone in particular is poorer in families with low incomes and this may lead to a delay in intervention for children at risk of ASD (Campbell et al., 2019). However, parents may also be led by their greater knowledge of the language domain to report what they think their child should be saying or understanding. For gesture, they have no accurate knowledge to mislead them, so they may report solely what they see their children doing, not what they expect their children to do. Tamis‐LeMonda et al. (2002) and Zand et al. (2015) found that young and lower income mothers were more likely to overestimate a children's abilities at a given age (including language ability). This could imply that some lower income/lower education groups may overestimate language competence in their infants. It is also true that anticipating milestones affects other parenting behaviours, beyond reporting their child's abilities (Hendrix & Thompson, 2011).

### Gesture‐language links in development

While a full discussion of the relationship between language and gesture development is beyond the scope of this paper, we have here assumed that gesture as measured in the UK‐CDI (W&G) is a parallel aspect of communication to word learning. The construction of the UK‐CDI (W&G) aimed to include communicative and symbolic aspects of gesture, and the literature to date back up this description of early gesture. For example, Iverson (2010) finds that early babble and early repetitive limb movements develop in parallel, and word‐gesture combinations appear before, and likely lead on to, two word combinations (Capirci et al., 1996; Iverson & Goldin‐Meadow, 2005). This close language/gesture link has been hypothesised to be due to the common symbolic nature of both language and gesture (Bates et al., 1989), but it could also be due to a common neural substrate for both types of motor skill (Alcock & Connor, 2021). We are not able to discriminate between these alternate explanations here, but it remains true that gesture is very important in communicative development, and potentially more easily and accurately measured.

### Future directions

The gesture scale of the UK‐CDI (W&G) should be useful for identifying children in categories that put them at risk for language delay, alongside the vocabulary production and comprehension scales of the UK‐CDI (W&G). We recommend that any future research on early communicative development includes gesture measures. Looking at the unexpected direction of some relationships between some risk factors and vocabulary scores, we can be surer that parents’ reports of gesture development accurately reflect children's true communicative abilities. parents’ reports of vocabulary development may depend on a more complex set of variables.

To examine further reasons for these unexpected directions we should in the future replicate this in another sample. We should also re‐examine the relationship between parent knowledge and parent reporting of language and gesture milestones across groups. Indeed, re‐analysis of the same dataset together with other similar datasets shows similar effects (Rowland et al., submitted). It is important that such a study should include sufficient proportions of parents with different age groups of mothers and different household income levels.

We also reported some important null findings: we did not find sex differences in vocabulary scores, nor any differences in language or communication scores between children whose mothers have differing levels of education. Looking at the former, while many studies find that girls’ language development is more advanced than boys, in some this difference is subtle (Fenson et al., 1994 finding the equivalent to 3 weeks of child age). In others it appears only at some age points, also finding a lack of difference late in the first year of life (Bornstein et al., 2004). Wallentin (2009) and Hyde and Linn (1988) conclude that, across the lifespan and including infants, sex differences in language ability have very small effect sizes. Bornstein et al. (2004) also found that there was no significant sex difference past the fifth year, suggesting this is due to different rates of maturation between the sexes, and that it may also explain other sex differences found in abilities (Galsworthy et al., 2000).

Looking at maternal education, this has been found in many analyses to be predictive of child language ability (Rudolph, 2017) but it is possible that culture‐specific or age‐specific factors are at play here. For example, only one of the studies in Rudolph's study was carried out in the UK, but the expansion of higher education provision in the UK finished in 2010 (Welch, 2021), when the dataset's youngest mothers would just have been entering university, leaving the older mothers less likely to have completed a degree, but on other measures likely to have better language outcomes for their children.

In future studies it will be important to compare cohorts with different levels of higher education, but large UK samples from past cohorts have not found very strong relationships between maternal education and children's language outcomes. The Avon Longitudinal Study of Children and Parents (ALSPAC) study (Law et al., 2019) found that a composite measure of general social disadvantage (not just maternal education), and a measure of proximal social disadvantage (the specifics of the household setting rather than parents’ characteristics) were the best predictors of language outcomes. Dale et al. (2015) suggested that SES (including maternal education) is mediated by caregiver input to affect child language, and that in their Twins’ Early Development Study, the effect of caregiver input was at least partly genetic. A further large international sample, including UK data, found no effect of SES (or child sex) on early language (Bergelson et al., 2023).

Future studies should also examine the predictive validity of the UK‐CDI (W&G) subscales: comprehension vocabulary, production vocabulary and gesture. Bavin and colleagues (Bavin et al., 2008; Reilly et al., 2006) found that gesture is a good predictor of later language from 12 months of age but not as good when measured at 8 months of age. Any future study should compare the predictive value from different ages.

### Summary and conclusions

Using an unprecedentedly large sample assessing children's gestures across a relatively wide infant age range and representative of the UK population as a whole, we found, in summary, that gesture may be a useful candidate for an early communication assessment toolbox, yet is often omitted. Its measurement is reliable and valid, and it appears to be linked to some biological risk factors that can lead children to have poor language and communication development at this early age. It also appears to have a more consistent relationship with some of the social risk factors that other studies have shown may put children at risk of poor language and communication development. Future research will have to show in more depth and detail if and how gesture interacts with comprehension and production. We conclude for now that it is not warranted to assess children's early vocabulary development at this age without adding an assessment of their early gesture development.

## CONFLICT OF INTEREST STATEMENT

There are no conflicts of interest, and this research is not part of a clinical trial.

## PARTICIPANT CONSENT

Ethics approval was granted for the original data collection exercise, and participants consented to the reuse of their data. No new data were collected for this publication.

## REPRODUCTION

Permission has been gained from John Wiley and Sons for reproduction of Figure 1a.

## Data Availability

The UK‐CDI (W&G) database which was analysed for this paper is available freely on the UK Data Archive, at https://reshare.ukdataservice.ac.uk/853687/. The study design was not preregistered.

## Items used in the Gesture Validation task

Communicative gestures

High five

Peekaboo

Clap hands

Symbolic gestures

Put phone to ear

Put on hat

Hold plane and make it fly

Comb or brush own hair

Put on glasses

Cover with blanket

Feed with spoon

## Items used in the Object Comprehension Task

ball

bike

bowl

chair

cheese

clock

cup

dress

duck

egg

fork

hat

horse

jumper

plane

plate

radio

soap

sock

stone
